# IDH2 mutation accelerates TPO‐induced myelofibrosis with enhanced S100a8/a9 and NFκB signaling in vivo

**DOI:** 10.1002/jha2.983

**Published:** 2024-07-28

**Authors:** Chien‐Chin Lin, Chi‐Yuan Yao, Yu‐Hung Wang, Yueh‐Chwen Hsu, Chang‐Tsu Yuan, Tsung‐Chih Chen, Chia‐Lang Hsu, Sze‐Hwei Lee, Jhih‐Yi Lee, Pin‐Tsen Shih, Chein‐Jun Kao, Po‐Han Chuang, Yuan‐Yeh Kuo, Hsin‐An Hou, Wen‐Chien Chou, Hwei‐Fang Tien

**Affiliations:** ^1^ Department of Laboratory Medicine National Taiwan University Hospital Taipei Taiwan; ^2^ Division of Hematology Department of Internal Medicine National Taiwan University Hospital Taipei Taiwan; ^3^ Graduate Institute of Clinical Medicine, College of Medicine National Taiwan University Taipei Taiwan; ^4^ Department of Pathology Graduate Institute of Oncology College of Medicine National Taiwan University Taipei Taiwan; ^5^ Department of Pathology National Taiwan University Cancer Center Taipei Taiwan; ^6^ Division of Hematology and Medical Oncology Department of Internal Medicine Taichung Veterans General Hospital Taipei Taiwan; ^7^ Department of Medical Research National Taiwan University Cancer Center Taipei Taiwan; ^8^ Division of Cellular Therapy Department of Integrated Diagnostics and Therapeutics National Taiwan University Hospital Taipei Taiwan; ^9^ Tai‐Cheng Stem Cell Therapy Center National Taiwan University Taipei Taiwan; ^10^ Division of Hematology and Medical Oncology Department of Internal Medicine Far Eastern Memorial Hospital New Taipei Taiwan

**Keywords:** IDH2 mutation, myelofibrosis, S100a8/a9, TPO

## Abstract

**Introduction:**

*IDH2* mutation is an unfavorable prognostic factor in patients with primary myelofibrosis (PMF) but its effect on myelofibrosis (MF) remains largely unclear.

**Methods:**

In this study, we aimed to elucidate the roles of *IDH2* mutation in the development and progression of MF by transcriptomic and molecular techniques using the *Idh2* ^R172K^ transgenic mice.

**Results:**

We found that thrombopoietin (TPO)‐overexpressed *Idh2* ^R172K^ (*Idh2* ^R172K^ + TPO) mice had accelerated progression to MF, compared with TPO‐overexpressed *Idh2‐*wild (WT + TPO) mice, showing activation of multiple inflammatory pathways, among which nuclear factor κB (NFκB) was the most significantly enhanced. Single‐cell transcriptomes of the marrow cells in early MF showed that *S100a8/a9* expression was mainly confined to neutrophil progenitors in the WT + TPO mice, but highly expressed in several types of myeloid precursor cells, including the megakaryocyte progenitors in the *Idh2* ^R172K^ + TPO group. Furthermore, *Idh2* ^R172K^ mice at age of 18 months had larger spleens, increased *S100a8/a9‐Tlr4* expression, and elevated serum S100a8/a9 levels compared with WT mice. PMF patients with *IDH2* mutations had higher bone marrow plasma S100A8/A9 levels than those without *IDH2* mutations.

**Conclusion:**

Overall, our findings showed that *IDH2* mutation induced proinflammatory effects, which further exacerbated MF, as evidenced by the increase in S100a8/a9 levels and NFκB hyperactivation in *Idh2* ^R172K^ + TPO mice.

## 1 INTRODUCTION

Philadelphia chromosome (Ph)‐negative myeloproliferative neoplasms (MPNs) are rare hematologic disorders characterized by clonal expansion of myeloid cells, constitutional symptoms, and inherent risk of progression to myelofibrosis (MF) and leukemia [1]. Classic MPNs consist of three subtypes: polycythemia vera (PV), essential thrombocythemia (ET), and primary myelofibrosis (PMF) [1]. PMF is further divided into pre‐PMF and overt‐PMF according to the fibrosis severity in the bone marrow (BM). PV and ET patients are at risk of developing post‐PV or post‐ET secondary MF after a long period of time. Patients with MF have the worst prognosis and highest risk of acute myeloid leukemia (AML) transformation among classic MPNs [1, 2].

MPNs arise from somatic mutations of three important and mutually exclusive driver genes: *JAK2*, the chaperone calreticulin (*CALR*), and the thrombopoietic receptor *MPL* in hematopoietic stem cells (HSC). These mutations activate several signals through receptors of erythropoietin, thrombopoietin (TPO), and granulocyte/macrophage colony‐stimulating factor, leading to constitutive activation of JAK2‒STAT signaling, myelo‐proliferation, cytokine overproduction, and subsequent MF [1].

In addition to the well‐known JAK‒STAT pathway, activation of the nuclear factor κB (NFκB) pathway was recently identified as a vital mechanism involved in BM inflammation in MF [3, 4, 5]. A recent study showed that compared with JAK inhibition alone, dual inhibition of JAK‒STAT and NFκB pathways could more effectively prevent disease progression and might even reverse fibrosis in BM [4]. NFκB signaling hyperactivation is induced by a combination of cell‐autonomous and non‐cell‐autonomous pathways following JAK‒STAT activation and cytokine stimulation, including tumor necrosis factor (TNF, also known as TNF‐α), interleukin‐1 (IL‐1) and IL‐6, and Toll‐like receptor (TLR) ligands, such as the alarmin heterocomplex S100A8/A9 [6]. Dysregulation of these pathways induces myeloproliferative disorder in BM in all MPN patients regardless of the mutation type [1]. In MPNs, inflammatory cytokines are responsible for the highly deleterious pathophysiological process in the BM microenvironment, suggesting that MPN is “a human inflammation model for cancer development.” However, MPN is considered more of an inflammatory disease than cancer [3, 6, 7].

Recently, high‐throughput genomic analyses of MPN patients have identified somatic mutations other than the three driver mutations [1, 8]. Among the mutations, *ASXL1*, *EZH2*, *SRSF2, U2AF1*, and *IDH1/2* mutations were associated with poor outcomes in PMF patients in Western patient cohorts [9, 10, 11, 12]. Notably, *IDH2*, but not *IDH1* mutations were identified as high‐risk factors in PV and ET patients [13].

*IDH1* and *IDH2* are recurrently mutated in myeloid neoplasms, including myelodysplastic syndrome (5%‒10%), acute myeloid leukemia (10%‒20%), and MPN (1%‒5%), as well as in lymphoma and some solid cancers [14, 15, 16, 17, 18]. Mutant IDH produces R‐2‐hydroxyglutarate (2HG), which induces histone and DNA hypermethylation through inhibition of epigenetic regulators [19]. In AML, 2HG induces DNA hypermethylation and leukemia formation, at least partially, by inhibiting the TET family of enzymes, which are involved in the first step of DNA demethylation [17].

Despite these data, the mechanism by which *IDH2* mutations accelerate MF progression remains unknown. Therefore, this study aimed to elucidate the role of *IDH2* mutation in the pathogenesis of MF using transcriptomic and molecular techniques. To achieve this, we induced MF in mice by overexpressing TPO in *Idh2‐*wild (WT) and *Idh2* R172K‐mutated (*Idh2* ^R172K^) HSC, followed by a detailed examination of hematopoietic phenotypes.

## 2 MATERIALS AND METHODS

### 2.1 Generation of *Idh2* ^R172K^ mice

*Idh2* ^R172K^ transgenic mice were generated for the experiments as described in our previous study [20]. Briefly, we introduced an AGG → AAG point mutation into exon 4 of the murine *Idh2* locus, leading to the R172K mutation (*Idh2* ^R172K^). Mice were housed in a specific pathogen‐free animal facility, and the study was approved by the Institutional Animal Care and Use Committee (IACUC) of National Taiwan University College of Medicine (IACUC approval number: 20210425).

### 2.2 Retroviral transduction of TPO in mouse BM cells

WT and *Idh2* ^R172K^ mice (8‒12 weeks old) were intraperitoneally injected with 150 mg/kg 5‐fluorouracil (Merck) 3 days before BM harvesting. Retrovirus constructs carrying TPO tagged with green fluorescence (GFP) were transfected into a Plat‐E retroviral packaging cell line (Cell Biolabs) 2 days before viral transduction. On the day of viral transduction, the virus concentrate was applied to the bone marrow cells (BMC) harvested from donor mice cultured in medium with cytokines as previously described [21].

### 2.3 Retroviral transduction of WT IDH and IDH^R172K^ in SET‐2 cell line

The SET‐2 cell line was purchased from DSMZ (ACC 608). A retrovirus construct carrying WT *IDH2* or *IDH* ^R172K^ tagged with GFP was transfected into a Plat‐A retroviral packaging cell line (Cell Biolabs) 2 days before viral transduction. The virus concentrate was applied to the SET‐2 cell line cultured in RPMI medium containing 20% fetal bovine serum (FBS).

### 2.4 Fluorescence‐activated cell sorting and analysis

GFP‐positive (GFP^+^) cells were sorted and analyzed using a FACSLSRII (BD Bioscience)/Attune (Thermo Fisher) at the Flow Cytometric Analyzing and Sorting Core Facility at the National Taiwan University Hospital (NTUH).

### 2.5 BM transplantation assay

GFP^+^ viable BMC were sorted 72 h after viral transduction and transplanted into lethally irradiated recipient mice as previously described [21]. B6.SJL‐Ptprc^α^pepc^β^/ BoyJ (CD45.1) recipient mice received a single lethal dose of 10 Gy irradiation followed by retro‐orbital injection of 100,000 GFP^+^ donor cells along with 10^5^ CD45.1 helper BMC. We checked the hemogram monthly after transplantation of the mice and sacrificed them at 12 weeks post‐transplant for further histologic and transcriptomic studies. For those used to explore the single‐cell transcriptomes of early MF, mice were sacrificed 4 weeks post‐transplant.

### 2.6 Tissue sectioning and immunohistochemistry staining

The spleens and femurs of the mice were excised, cleaned, and embedded in paraffin after being humanely sacrificed under anesthesia. Sections (4 µm) were stained with hematoxylin and eosin (H&E), reticulin, and Masson's trichrome. Phospho NFκB p65 (Bioss) and MRP8 (S100A8) antibodies (Abcam) were used for activated NFκB and S100a8 immunohistochemistry (IHC) staining, respectively.

### 2.7 Analysis of the gene expression profile

Raw reads were aligned to the mouse reference genome GRCm38, and gene‐level expression was quantified in expected counts and transcripts per kilobase million (TPM) using RNA‐Seq by Expectation Maximization (RSEM) software. The RSEM expected counts were normalized using the trimmed mean of the *M*‐values method implemented in the R package edgeR. Differentially expressed genes (DEGs) were identified using the Wilcoxon rank sum test. Genes that were detected in more than 25% of a specific cluster were included in the DEG analysis, with the minimum fold‐change threshold set to 1.25. Gene set enrichment analysis (GSEA) was implemented in the R package clusterProfiler [22] and the gene sets used for analysis were downloaded from MSigDB (https://www.broadinstitute.org/gsea/msigdb/). Genes were pre‐ranked in the order of differential expression between the conditions [21, 23]. Protein‒protein interaction network analysis was performed using STRING database v11.5 (available at: http://string‐db.org/) [24]. We filtered for the interactions belonging to *Mus musculus* to grow the interaction network and further refined it to include only those interactions connecting S100a8 and S100a9 with confidence scores >0.5.

### 2.8 Single‐cell RNA sequencing data pre‐processing and analysis

Single‐cell RNA sequencing (scRNA‐seq) library preparation and analysis were performed as previously described [20]. Approximately 15,000 lineage‐negative BM cells from mice were FACS sorted into 20 µL Dulbecco's Modified Eagle Medium (DMEM)/10% FBS and then loaded onto the 10× chromium controller, according to the manufacturer's instructions (Supporting Information).

Lineage assignment of cell clusters was performed by inspecting the expression patterns of canonical lineage‐specific markers that have been previously reported in the literature on uniform manifold approximation and projection (UMAP) space [25, 26, 27].

### 2.9 PMF patient cohort

We extended the PMF patient cohort from the NTUH with clinical information, including the results of cytogenetics and mutations detected by targeted next‐generation sequencing, as previously mentioned [11, 28]. Institutional Review Board (IRB) of NTUH approved the collection of patients’ BM samples for further studies (IRB approval number: 201709072RINC) with informed consent from all subjects.

### 2.10 Enzyme‐linked immunosorbent assay for human S100A8/A9 and mouse S100a8/a9

S100A8/A9 levels in the BM plasma of MF patients and S100a8/a9 levels in the serum of mice were determined using the human S100A8/S100A9 Heterodimer Quantikine (Bio‐Techne, R&D) and mouse S100A8/S100A9 Heterodimer DuoSet (Bio‐Techne, R&D) enzyme‐linked immunosorbent assay (ELISA) kits, according to the manufacturer's instructions.

### 2.11 Statistical analysis

Data were processed using GraphPad Prism (GraphPad Software) or Microsoft Excel (Microsoft), and differences between means were determined using Student's *t*‐test. Means were considered significant at *p *< 0.05.

## 3 RESULTS

### 3.1 *Idh2* ^R172K^ mutation accelerates MF progression in the TPO mouse model

**FIGURE 1:**
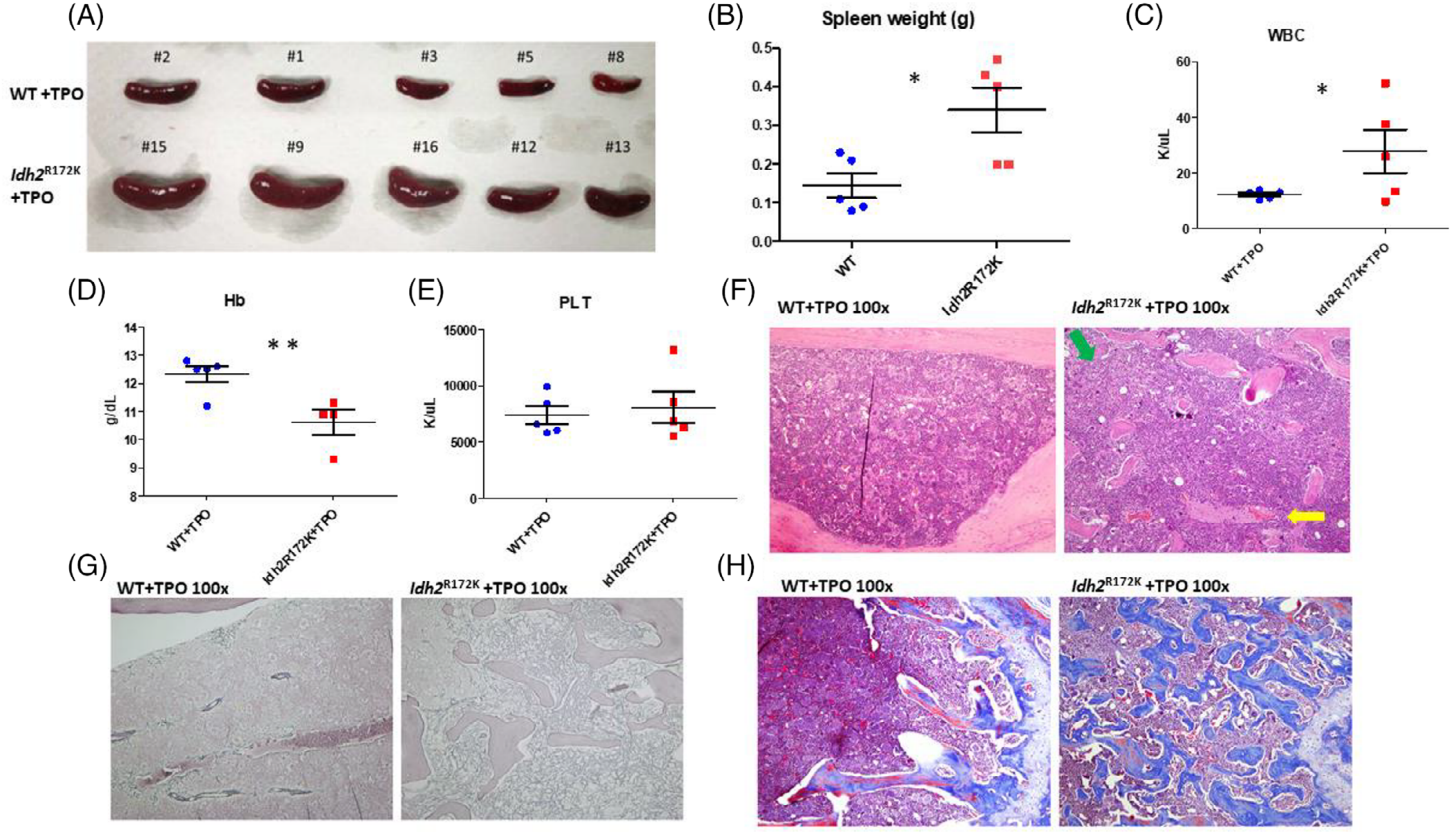
*Idh2* ^R172K^ + thrombopoietin (TPO) mice had more severe myelofibrosis (MF) phenotypes than wild‐type (WT) + TPO mice. (A) Representative images of the spleens of mice sacrificed at 12 weeks post‐transplant. Upper: WT + TPO (*n* = 5); lower: *Idh2* ^R172K^ + TPO (*n* = 5). (B) Spleen weight of mice in the two groups (*n* = 5 in each group). (C‒E) Hemogram of the two groups of mice at 12 weeks post‐transplant: (C) white blood cells (WBC), (D) hemoglobin, and (E) platelets (*n* = 5 in each group). *Idh2* ^R172K^ + TPO mice had significantly higher WBC counts and lower hemoglobin levels than the control group. (F) Bone marrow (BM) sections showed increased megakaryocytes in both groups, consistent with the TPO‐related myeloproliferative neoplasm (MPN) phenotype. Cellular streaming (green arrow) and dilated sinusoids with intra‐sinusoidal hematopoiesis (yellow arrow) were noted in the *Idh2* ^R172K^ + TPO group, implying a more severe MF phenotype (original magnification ×100). (G) Reticulin staining revealed significantly increased reticulin in the *Idh2* ^R172K^ + TPO group (grade 3) compared with the WT + TPO group (grade 1) (original magnification ×100). (H) Masson's trichrome staining showed collagenous fibers and significant osteosclerosis in the *Idh2* ^R172K^ + TPO group compared with the control group (original magnification ×100). Significant differences were determined using a *t*‐test; ^*^ *p* < 0.05, ^**^ *p* < 0.01.

Thrombopoiesis was induced in WT and *Idh2* ^R172K^ transgenic mice [20] to evaluate the effects of *IDH2* mutations in MF [29, 30, 31]. We overexpressed *TPO* in WT and *Idh2* ^R172K^ mouse marrow cells, followed by transplantation into syngeneic mice (designated as WT + TPO and *Idh2* ^R172K^ + TPO hereafter). *Idh2* ^R172K^ + TPO mice had larger spleens (Figure 1A,B), lower hemoglobin levels (*p* < 0.01), and higher white cell counts (*p *< 0.05) than WT + TPO mice 12 weeks after transplantation; however, there was no significant difference in platelet count between the groups (Figure 1C‒E). H&E and reticulin staining of the BM sections showed grade 2‒3 fibrosis in most *Idh2* ^R172K^ + TPO mice, whereas all WT + TPO mice had only grade 1 fibrosis (Figure 1F,G). Masson's trichrome staining confirmed severe marrow fibrosis with positive staining of collagen in the *Idh2* ^R172K^ + TPO mice (Figure 1H), with some mice developing prominent osteosclerosis with increased formation and thickening of the trabecular bones (Figure 1H).

### 3.2 NFκB pathways were hyperactivated in young *Idh2* ^R172K^ + TPO mice

**FIGURE 2:**
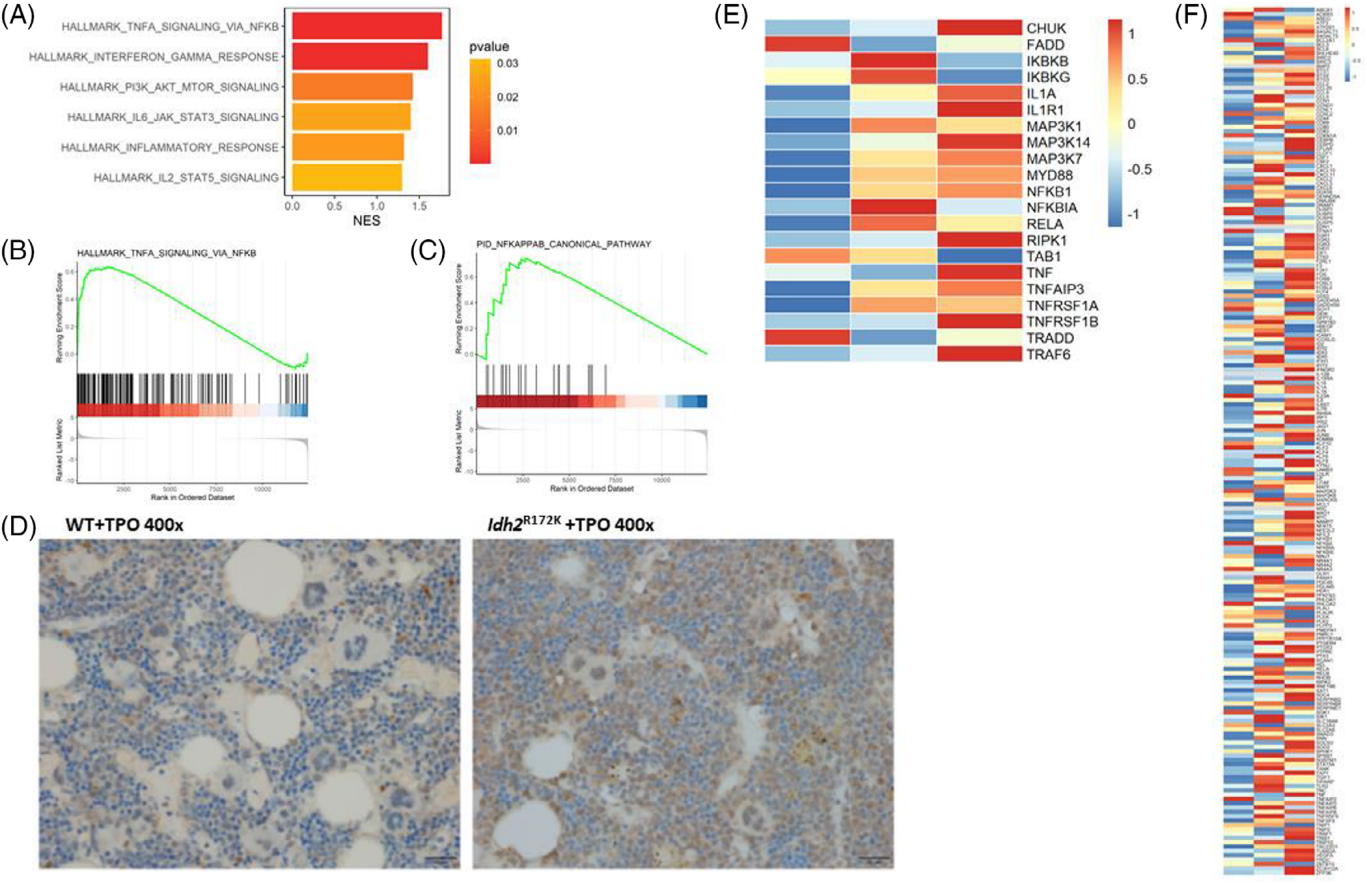
Nuclear factor κB (NFκB) pathways were hyperactivated in *Idh2* ^R172K^ + thrombopoietin (TPO) mice and IDH2^R172K^ + Jak2 V617F SET‐2 cells. (A) Gene set enrichment analysis (GSEA) revealed that several inflammatory signaling pathways were significantly enriched in the *Idh2* ^R172K^ + TPO group, including tumor necrosis factor (TNF‐α) signaling via NFκB, interferon gamma response, and IL2 stat5, PI3K_Akt_Mtor, and IL6 Jak_Stat3 signaling. NFκB pathways including TNF‐α signaling via NFκB (B) and NFκB canonical pathway (C) were significantly upregulated in the *Idh2* ^R172K^ + TPO group (*p *< 0.001 and <0.05). (D) Immunohistochemistry (IHC) staining for phosphor‐P65 showed strong nuclear staining of megakaryocytes and myeloid cells, consistent with the hyperactivated NFκB signaling in the *Idh2* ^R172K^ + TPO group (original magnification ×400). (E and F) SET‐2 cells transduced with IDH2^R172K^ had hyperactivated NFκB signaling including NFκB canonical pathway (E) and TNF‐α signaling (F) compared to wild‐type *IDH2* and empty vectors. Left: SET‐2 cells transduced with empty vector; middle: wild *IDH2*; right: *IDH2* ^R172K^.

The transcriptomes of whole BM cells from three pairs of WT + TPO and *Idh2* ^R172K^ + TPO mice sacrificed at 12 weeks post‐transplantation were compared to elucidate the mechanism that *IDH2* mutation enhanced MF. Multidimensional scaling revealed distinct global gene expression patterns between the two groups of mice (Figure S1). GSEA revealed that several inflammatory signaling pathways were significantly upregulated in the *Idh2* ^R172K^ + TPO group, including TNFα signaling via NFκB, interferon gamma response, and IL2_stat5, PI3K_Akt_Mtor, and IL6_Jak_Stat3 signaling (Figure 2A). Notably, NFκB pathways, including TNFα signaling via the NFκB canonical pathway, were markedly upregulated in the *Idh2* ^R172K^ + TPO group (Figure 2B,C).

BM sections were IHC stained for phospho‐p65 expression, a component of the activated NFκB complex, to confirm the functional activation of NFκB in *Idh2* ^R172K^ + TPO mice. Consistent with the transcriptome data, phospho‐p65 was highly expressed in the nuclei of multiple cell types, including hematopoietic stem and progenitor cells (HSPC), megakaryocytes, and other myeloid cells of the BM of *Idh2* ^R172K^ + TPO mouse compared with the WT + TPO mice (Figure 2D).

### 3.3 NFκB pathways were hyperactivated in IDH2^R172K^‐transduced SET‐2 cells

To further examine the direct effects of *IDH2* mutation on megakaryocytic progenitors, we overexpressed WT *IDH2* and *IDH2^R172K^* in SET‐2 cell line, a human megakaryoblast cell line originating from an ET patient harboring an inherent *JAK2*V617F mutation [32]. Transcriptome analysis revealed that NFκB signaling was hyperactivated in *IDH2^R172K^*‐transfected SET‐2 cells compared with cells transfected with WT *IDH2* and empty vectors (Figure 2E,F), indicating that *IDH2* mutation can activate NFκB under the background of JAK‒STAT pathway activation.

### 3.4 Hyperactivated S100a8/a9 expression and NFκB signaling in *Idh2* ^R172K^ + TPO myeloid progenitors during early MF

**FIGURE 3:**
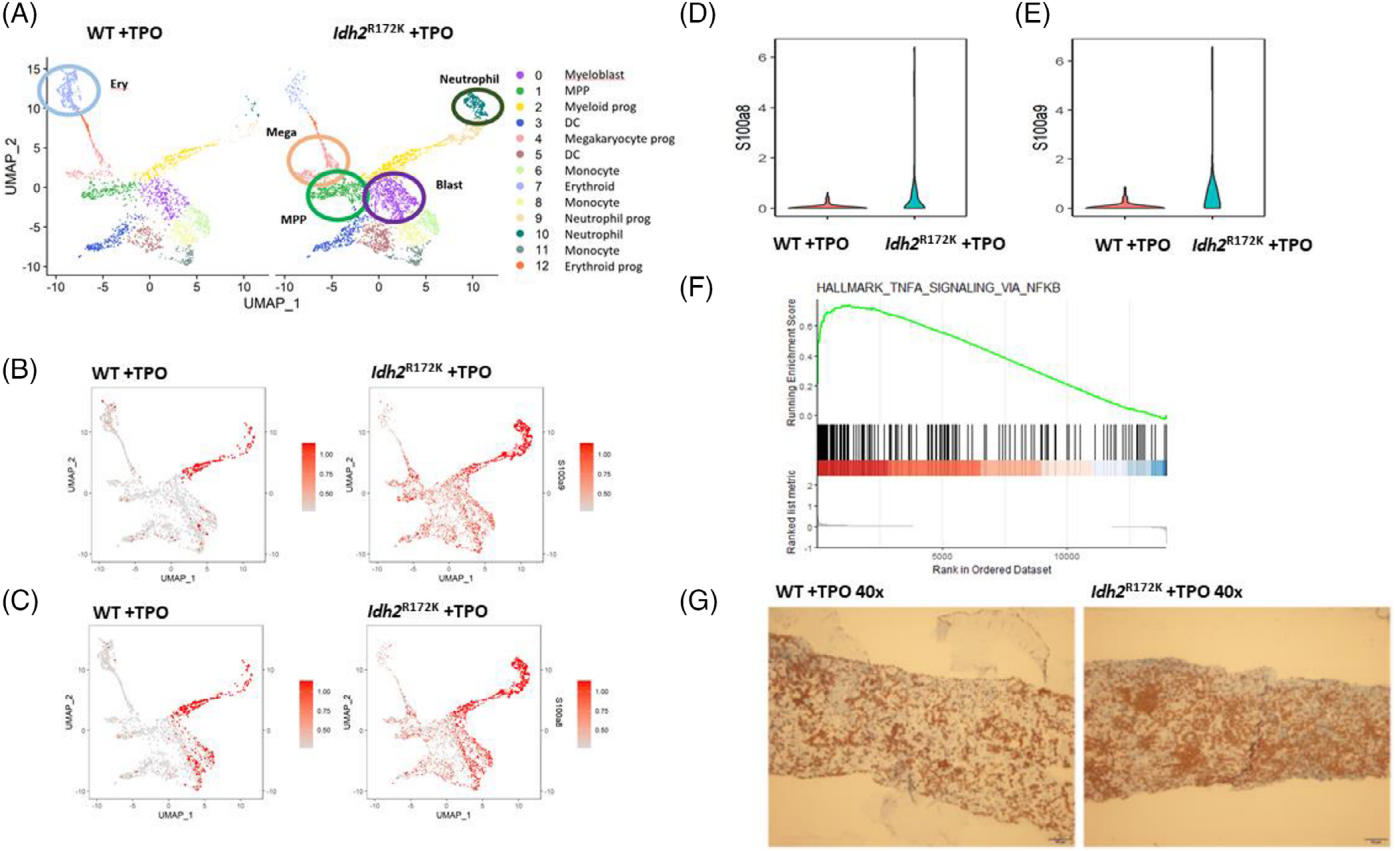
Single‐cell transcriptome profiling of bone marrow cells (BMCs) from *Idh2‐*wild (WT) + thrombopoietin (TPO) and *Idh2* ^R172K^ + TPO mice in earlier stage of myelofibrosis (MF) formation (4 weeks post‐transplant). (A) Uniform manifold approximation and projection (UMAP) representation of single cells from the WT + TPO and *Idh2* ^R172K^ + TPO mice Lin‐BMC, classified into 12 distinct clusters. Cells were annotated by the expression pattern of canonical lineage‐specific markers, according to previously described methods. (B) *S100a8* expression levels in various subpopulations. (C) *S100a9* expression levels in various subpopulations. (D) Violin plots of normalized expression of *S100a8* in Meg_prog. The level was significantly higher in the *Idh2* ^R172K^ + TPO group (*p *< 0.001). (E) Violin plots illustration of normalized expression of *S100a9* in Meg_prog. The level was significantly higher in the *Idh2* ^R172K^ + TPO group (*p *< 0.001). (F) Gene set enrichment analysis (GSEA) showed upregulation of tumor necrosis factor (TNF‐α) signaling via nuclear factor κB (NFκB) signaling (*p *< 0.001) in Meg_prog cells in *Idh2* ^R172K^ + TPO mice. (G) Immunohistochemistry (IHC) staining revealed an increase in S100a8 expression in the *Idh2* ^R172K^ + TPO group (original magnification ×100).

scRNA‐seq of pre‐fibrotic Lin^−^ BM cells from mice (4 weeks post‐transplantation) was performed to further elucidate the pathogenesis of MF. We focused on myeloid lineages and projected the cells onto the UMAP after removing batch effects and aligning cells from two pairs of samples (WT + TPO vs. *Idh2* ^R172K^ + TPO). Cell types included myeloblast, multipotent progenitor, myeloid progenitor (Mye_Prog), megakaryocyte progenitor (Meg_Prog), erythroid progenitor (Ery_Prog), erythroid cell (Ery), neutrophil progenitor (Neu_Prog), and various subpopulations of monocytes and dendritic cells. Compared with WT + TPO group, the *Idh2* ^R172K^ + TPO group had higher percentages of HSPC, Meg_Prog, and neutrophils, but a lower percentage of erythroid cells (Figure 3A), suggesting a more advanced disease stage [33]. The inflammatory alarmins *S100a8* and *S100a9* were among the top DEGs (Table S1) in various myeloid cells in *Idh2* ^R172K^ + TPO mice, including mature and immature populations. In contrast, their expression was confined to neutrophils and neutrophil progenitors in WT + TPO mice (Figure 3B,C).

Since megakaryocytes are critical for MF progression [34], we focused on the Meg_Prog population. There was a significant increase in *S100a8* and *S100a9* expression in Meg_Prog in the *Idh2* ^R172K^ +TPO group compared with the WT + TPO group (Figure 3D,E). Moreover, GSEA indicated that the NFκB pathway was significantly enriched in the *Idh2* ^R172K^ + TPO group (Figure 3F), indicating the importance of S100a8/a9 and NFκB in MF progression in *Idh2* ^R172K^ mice. Furthermore, IHC staining confirmed higher S100a8 expression in the BM sections of *Idh2* ^R172K^ + TPO mice (Figure 3G).

### 3.5 Enhanced S100a8/a9 expression and inflammaging pathways in aged *Idh2* ^R172K^ mice

**FIGURE 4:**
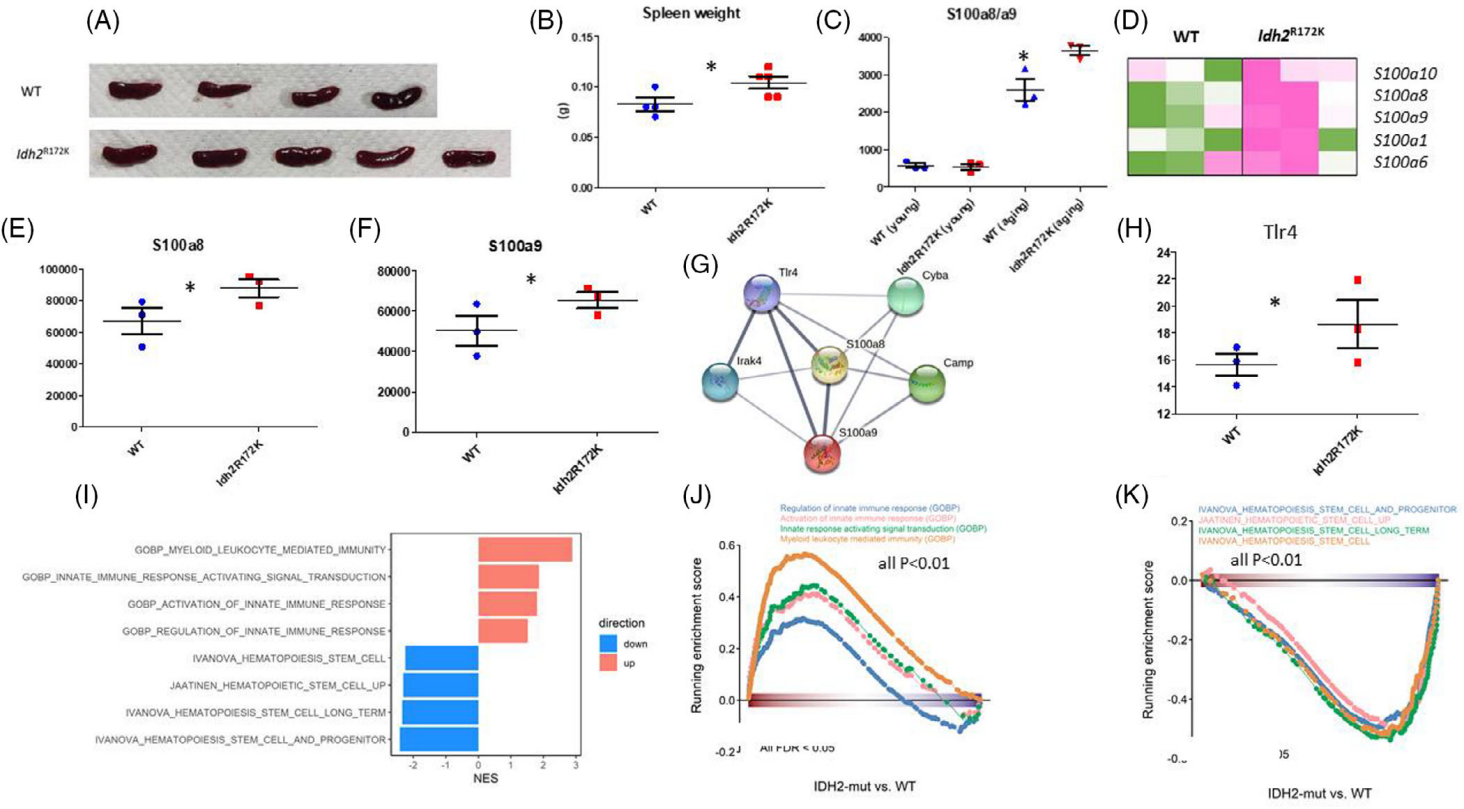
Aged *Idh2* ^R172K^ mice had higher alarmin S100a8/a9 expression and inflammaging phenotypes compared with age‐matched littermates. A *Idh2* ^R172K^ mice at 18 months old had larger spleens compared with age‐matched wild‐type (WT) littermates. Upper: WT (*n* = 4); lower: *Idh2* ^R172K^ (*n* = 5). (B) Determination of the spleen weight of mice in the two groups: WT (*n* = 4); *Idh2* ^R172K^ (*n* = 5). (C) Serum S100a8/a9 levels were not significantly different between young WT and *Idh2* ^R172K^ mice; however, aged *Idh2* ^R172K^ mice had significantly higher levels than their littermate controls (*n* = 3 in each group). (D) Expression levels of *S100a* family genes. Several *S100a* genes, including *S100a10*, *S100a8*, *S100a9*, *S100a1*, and *S100a6* were upregulated in the *Idh2* ^R172K^ group compared with the control group (*n* = 3 in each group), illustrated as heatmap. *S100a8* (E) and *S100a9* (F) expression levels were significantly higher in *Idh2* ^R172K^ mice compared with the control group (*n* = 3 in each group). (G) Protein interaction network analysis using STRING database specifically connected S100a8 and S100a9. The S100a8/a9 receptor Tlr4 was highly connected with S100a8 and S100a9 in the dataset, suggesting an activation of the S100a8/a9‐Tlr4 pathway in elderly *Idh2* ^R172K^ mice. (H) *Tlr4* expression was significantly higher in *Idh2* ^R172K^ mice compared with the control group (*n* = 3 in each group). (I‒K) Gene set enrichment analysis (GSEA) showed that pathways related with innate immunity were significantly upregulated, whereas hematopoietic stem and progenitor cell (HSPC) hematopoiesis pathways were downregulated (all *p* < 0.01), suggesting a proinflammatory status in aged *Idh2* ^R172K^ mice. Significant differences were determined using a *t*‐test; ^*^ *p* < 0.05.

PMF is an inflammatory disease prevalent in the elderly. Aging has been shown to trigger sterile inflammation (inflammaging), which involves NFκB and several innate immune pathways [35, 36]. Although there were no marked difference in terms of hemogram and BM histologic findings between the aged *Idh2* ^R172K^ and WT mice [20], significantly enlarged spleens were observed in aged (18 months) *Idh2* ^R172K^ mice compared with WT littermates (Figure 4A,B). Additionally, serum S100a8/a9 levels were significantly elevated in aged *Idh2* ^R172K^ mice compared with their littermate WT controls as well (Figure 4C). Consistent with these findings, several *S100* family genes, including *S100a10*, *S100a8*, *S100a9*, *S100a1*, and *S100a6*, were significantly upregulated in whole BM cells from aged *Idh2* ^R172K^ mice (Figure 4D‒F). Of note, *Tlr4*, which encodes a TLR Tlr4 protein as the S100a8/9 receptor, was significantly upregulated in the *Idh2* ^R172K^ mice (Figure 4G,H), indicating the activation of S100a8/a9 and TLR signaling pathways in *Idh2* ^R172K^ mice during aging. GSEA showed that several immune‐related pathways were activated, whereas HSC pathways were downregulated in aged *Idh2* ^R172K^ mice compared with aged WT mice. Overall, these data indicate a proinflammatory function for *Idh2* ^R172K^ mutation (Figure 4I‒K).

### 3.6 S100A8/A9 levels in BM plasma were higher in IDH2‐mutated MF patients

**FIGURE 5:**
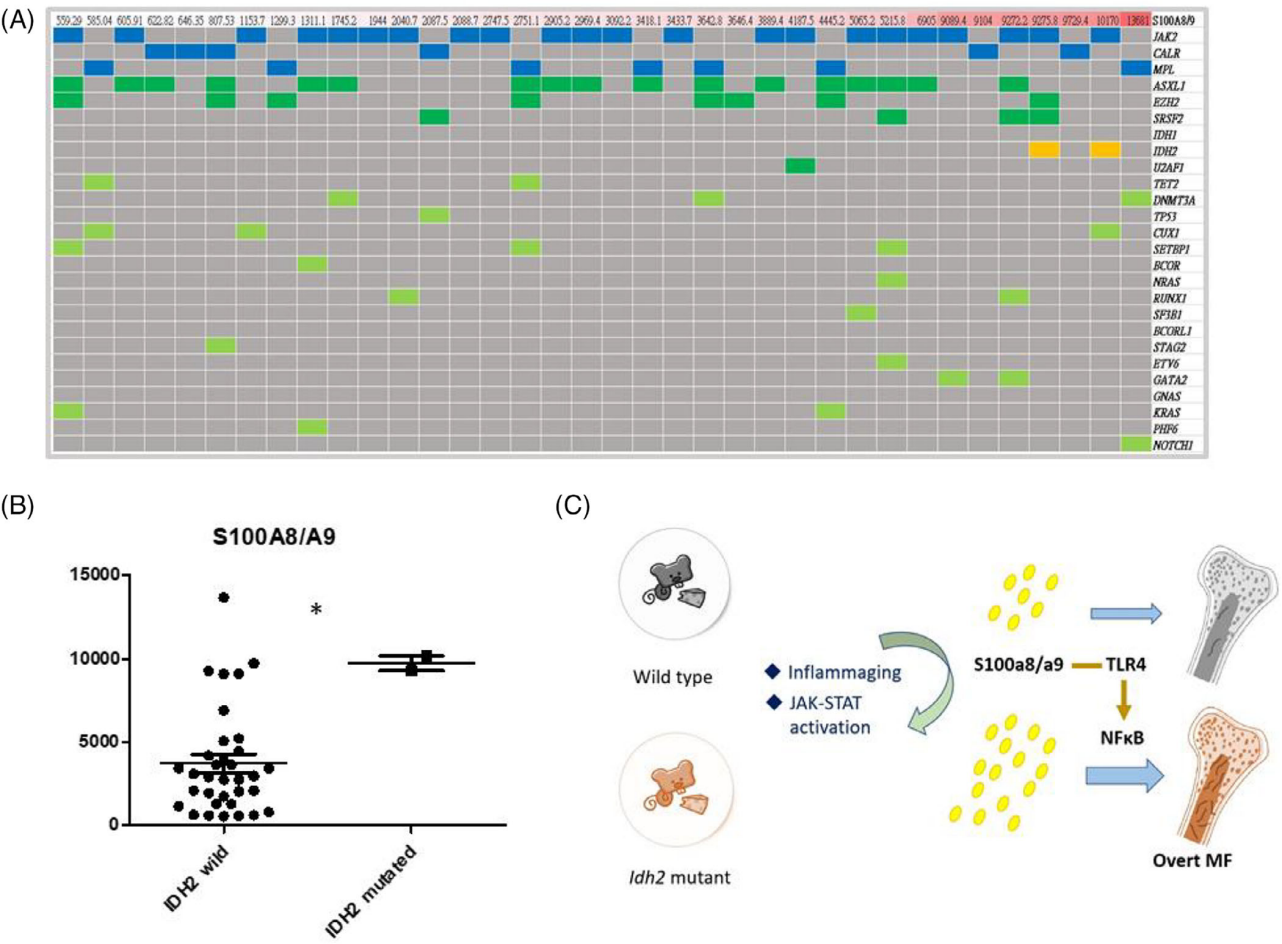
Higher expression of S100A8/A9 heterodimer in the bone marrow (BM) of *IDH2*‐mutated primary myelofibrosis (PMF) patients. (A) Mutation landscape in PMF patients. The profiles were sorted according to plasma S100A8/A9 expression (top bar), which was determined using enzyme‐linked immunosorbent assay (ELISA) kits (left to right, low to high). Driver mutations, blue color; high molecular risk (HMR) mutations, dark green color; *IDH2* mutation, yellow color; other mutations, light green color. (B) S100A8/A9 expression levels between PMF patients with (*n* = 2) and without (*n* = 34) *IDH2* mutation was illustrated using dot plots. S100A8/A9 expression was significantly higher in patients with *IDH2* mutation. (C) Graphic description of the summary of this study. Significant differences were determined using a *t*‐test; ^*^ *p* < 0.05.

From the findings in the mouse model of TPO‐induced MF shown above, it was suggested that *Idh2* mutation might increase S100A8/A9 levels. To verify this in humans, the S100A8/A9 levels of the BM plasma obtained from MF patients were determined using ELISA kit. Among the 36 patients with available BM plasma S100A8/A9 levels and targeted sequencing data, two harbored *IDH2* mutations with similar allele frequencies at 48.4% and 49.4%, respectively. The mutation profiles of the patients and their corresponding S100A8/A9 levels are shown in Figure 5A. The two *IDH2*‐mutated BM samples had significantly higher S100A8/9 expression levels than the other samples (Figure 5B). Overall, our study demonstrated that *Idh2* ^R172K^ ‐mutated BM cells produced more S100a8/a9 alarmin (a TLR4 ligand) during aging and JAK‒STAT activation, leading to the hyperactivation of NFκB pathway and more severe MF phenotypes in a TPO mouse model. A schematic illustration of these findings is shown in Figure 5C.

## 4 DISCUSSION

In MPN, hyperactivation of the JAK‒STAT pathway induces the proliferation of myeloid lineage cells and cytokines production, including IL‐1, IL‐6, IL‐8, and TNF, and the alarmin heterocomplex S100A8/A9 [6, 34]. Concurrent mutations other than the three major driver mutations (*JAK2*, *CALR*, *MPL* mutations) of JAK‒STAT pathway are associated with disease progression, including MF and AML transformation. Specifically, *IDH* mutations have been recognized as a poor prognostic factor of MF [11, 37, 38]. Moreover, *IDH2* mutations were identified as the only high‐risk mutations in ET, PV, and PMF in the Mayo‐Florence cohorts [10, 13]. Although *IDH* mutations are clinically relevant, their role in the pathogenesis of MPN remains unclear. McKenney et al. showed that mice with combined *Jak2* ^V617F^ and *IDH* mutations had higher levels of HSPC, and treatment with combined inhibitors normalized the HSPC levels and reversed aberrant gene expression in murine models. However, both double‐hit and single *JAK2*V617F mutant mice exhibited similar phenotypes [39]. In the present study, TPO‐overexpressed *Idh2* ^R172K^ mice exhibited severe MF, compared to TPO‐overexpressed *Idh2*‐wild mice, which was partially related to the hyperactivation of the NFκB pathway, as revealed by RNA‐seq and IHC staining of BM. We believe that the phenotypic discrepancies between the study by McKenney et al. and ours are mainly due to the different murine MF models used. All mice induced by TPO developed dominant MF within a few months [29, 40], while for McKenney et al., the phenotypes of the *JAK2*V617F mutant knock‐in mice are much more indolent [39].

The transcription factor NFκB serves as a pivotal mediator of inflammatory responses and links persistent chronic inflammation to an increased risk of solid cancers and hematologic malignancies, including lymphoid neoplasms, AML, MDS, and MF [5, 41–44]. In MF, NFκB hyperactivation is induced via both cell‐autonomous signaling downstream of the mutant JAK2 kinase and non‐cell autonomous activation by inflammatory mediators [5, 6, 45]. Fisher et al. showed that hyperactivation of NFκB was observed in the HSPC of MF patients, indicating its role in the development or maintenance of clonal dominance [5]. Moreover, other studies have shown that NFκB signaling hyperactivation is not confined to HSPC, but is also transmitted throughout both non‐malignant hematopoietic cells and BM stroma via inflammatory mediators, including IL‐1, TNF, and the TLR ligands S100A8/A9 [4, 6, 46]. Functionally altered mesenchymal stromal cell (MSC) further create a proinflammatory BM microenvironment that propagates malignant transformation of HSC but suppresses normal hematopoiesis, resulting in the vicious circle of inflammatory interaction between hematopoietic cells and MSC [3, 7, 47]. Inflammation mediators secreted from myeloid cells and MSC stimulate Lepr^+^ MSC and Gli1^+^ MSC to deposit extracellular matrix, leading to MF [7, 30, 31, 47]. The alarmin heterocomplex S100A8/A9 secreted by the BM has been reported as a candidate driver initiating the inflammatory crosstalk in myeloid neoplasm and MF [47, 48]. Leimkühler et al. showed that overproduction of S100A8/A9 by BM myeloid cells and megakaryocytes, followed by stroma cells plays a central role in MF progression through a self‐perpetuating cycle of the inflammation network between stromal cells and hematopoietic cells [34].

The impact of *IDH* mutations on the cytokine signaling response in HSPC remains largely unknown. In the present study, scRNA‐seq analysis showed that S100a8/a9 and NFκB signaling were significantly upregulated in TPO‐overexpressed *Idh2*‐mutated BM HSPC and megakaryocyte progenitors compared with the *Idh2*‐wild counterpart. Moreover, S100a8/a9 levels were also elevated in aged *Idh2*‐mutated mice, which is consistent with a sterile inflammatory phenotype. Additionally, transcriptome analysis revealed positively enriched innate immune pathways and negatively enriched HSPC pathways in aged *Idh2*‐mutated mice, which are the two hallmarks of inflammaging. These results are consistent with a recent report, in which *IDH2*‐mutated leukemia cells were highly sensitive to IL1β, with augmented response at multiple downstream targets of the NFκB pathway [49]. That study showed that *IDH2*‐mutated AML cell lines exhibited significantly higher sensitivity to IL‐1β across various downstream readouts. Additionally, bulk RNA sequencing revealed elevated cytokine‐related signaling pathways and NFκB target genes. scRNA‐seq of both unstimulated and stimulated cells confirmed modified IL‐1β transcriptional responses in the *IDH2*‐mutant cells. Targeted inhibition of the IκB kinase complex reduced IL‐1β responses and induced cell death in primary *IDH*‐mutated leukemia samples [49].

Recent studies have shown that MSCs in the BM also play important inflammatory roles in the BM [34]. In *IDH*‐mutated AML, 2HG activates the NFκB pathway in BM stromal cells, leading to the production of cytokines, including IL‐6, IL‐8, and complement 5a, which might stimulate the progression of AML [50]. The pathological roles of BM stromal cells in *IDH*‐mutated MPN need to be explored in the future. In summary, our study revealed the important roles of the alarmin heterocomplex S100A8/A9 in *Idh2‐*mutated mice during aging and early MF formation, providing new insights for further mechanistic and therapeutic exploration.

## AUTHOR CONTRIBUTIONS

Chien‐Chin Lin wrote the manuscript, designed the study, performed the experiments, and analyzed the data. Chi‐Yuan Yao, Yu‐Hung Wang, and Chia‐Lang Hsu performed the bioinformatics analyses. Chang‐Tsu Yuan interpreted tissue sections. Sze‐Hwei Lee, Yueh‐Chwen Hsu, Tsung‐Chih Chen, Jhih‐Yi Lee, Pin‐Tsen Shih, Chein‐Jun Kao, Po‐Han Chuang, and Yuan‐Yeh Kuo performed experiments. Hsin‐An Hou performed the clinical data collection. Hwei‐Fang Tien organized and revised the manuscript. Wen‐Chien Chou provided the concept, coordinated the study, and revised the manuscript accordingly.

## CONFLICT OF INTEREST STATEMENT

The authors declare they have no conflicts of interest.

## ETHICS STATEMENT

The Institutional Review Board (IRB) of NTUH approved the study (IRB approval number: 201709072RINC).

## PATIENT CONSENT STATEMENT

The Institutional Review Board (IRB) of NTUH approved the study (IRB approval number: 201709072RINC) with informed consent from all subjects.

## CLINICAL TRIAL REGISTRATION

The authors have confirmed that clinical trial registration is not needed for this submission.

## PERMISSION TO REPRODUCE MATERIAL FROM OTHER SOURCES

The article did not reproduce material from other sources.

## Supporting information

Supporting Information

## Data Availability

The data that support the findings of this study are available from the corresponding author upon request.
