# Supplementary material for: IDH2 mutation accelerates TPO‐induced myelofibrosis with enhanced S100a8/a9 and NFκB signaling in vivo
Source: EJHaem. 2024 Jul 28;5(4):738–48. doi: 10.1002/jha2.983 (PMC11327712; doi:10.1002/jha2.983)
Supplement: Supplementary file 1 — Supporting Information [file JHA2-5-738-s001.docx]

**Supplementary information**

**Methods**

**Next generation sequencing**

The mRNA was purified from total RNA (1 µg) by oligo(dT)-coupled magnetic beads and fragmented into small pieces under elevated temperature. The first-strand cDNA was synthesized using reverse transcriptase and random primers. After the generation of double-strand cDNA and adenylation on 3’ ends of DNA fragments, the adaptors were ligated and purified with AMPure XP system (Beckman Coulter, Beverly, USA). The quality of the libraries was assessed on the Agilent Bioanalyzer 2100 system and real-time PCR. The qualified libraries were sequenced on an Illumina NovaSeq 6000 platform with 150 bp paired-end reads generated by Genomics, BioSci & Tech Co., New Taipei City, Taiwan.

**Single-cell sequencing**

The scRNA-seq libraries in this study were prepared following the

Chromium Single Cell 3' Reagent Kits User Guide. Around 15,000 lineage-negative BM cells from mice of the two samples (WT+TPO, *Idh2*^R172K^+TPO) were FACS-sorted into 20 μL DMEM/10% FBS and then loaded onto the 10x Chromium Controller according to the recommendations of the manufacturer. The samples were processed with the Chromium Next GEM Single Cell 3’ Library Construction Kit v3.1 (10X Genomics, Pleasanton, CA, US), according to the protocol. The cells were loaded onto the chip of the Chromium Controller for droplet formation and reverse transcription. The multiplexed cDNA libraries were sequenced on NextSeq 500 with the goal of obtaining more than 20,000 reads per cell.

**10x Genomics single-cell RNA sequencing data pre-processing and analysis**

Sequencing data were processed with the Cell Ranger software (version 3.0.1) from 10x Genomics. FASTQ reads were aligned to the mouse reference genome (GRCm38) to obtain unique molecular identifier (UMI) counts and to generate the expression matrix for each library. The cells for analysis met the following quality control (QC) thresholds: UMI counts > 1,000 and ≤ 50,000); number of detected genes > 500 and ≤ 5,500); percentage of mitochondrial gene expression < 10% per cell; and genes included in analysis were those expressed in at least 10 cells. The Seurat software (version 3.0.0) was used for the further analysis. (PMID: 29608179) The feature expression measurements of each library were scaled to the size of 10,000, and then log transformed. The highly variable genes were identified on the log-transformed data. The data from each library were integrated for the correction of technical differences between samples. Principal components analysis (PCA) was performed using highly variable genes, and the dimensionality of the dataset was estimated by the elbow plot. Cells were then projected on the uniform manifold approximation and projection (UMAP) space using the first 30 principal components of the data matrix. Cell clustering was performed using the Louvain graph-based clustering method, with the resolution parameter set to = 0.3, which resulted in thirteen major clusters representing HSPC and myeloid populations.

**Supplementary figure 1.**

Multidimensional scaling showed distinct global gene expression patterns between WT+TPO and *Idh2*^R172K^ +TPO mice sacrificed at 12 weeks post-transplant.


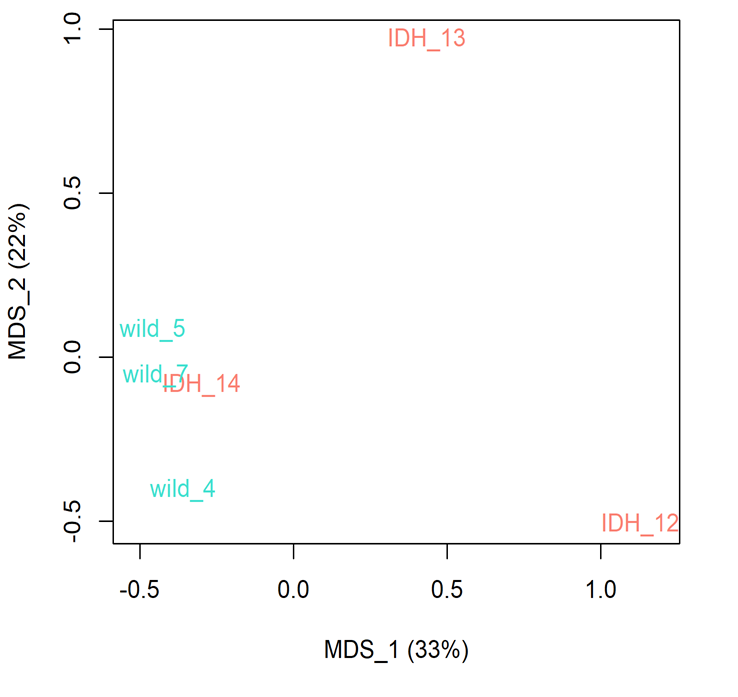


**Supplementary table 1.**

Top 20 differentially expressed genes in Blast (a), MPP (b) and Meg_prog (c).

*S100a8* and *S100a9* were among the top upregulated genes.

(a)

(b)

(C)
